# Chemical Examination of *Citrus sinensis* Flavedo Variety Pineapple

**DOI:** 10.4103/0250-474X.59552

**Published:** 2009

**Authors:** Geeta Rani, Lalita Yadav, S. B. Kalidhar

**Affiliations:** Department of Chemistry and Physics, CCS Haryana Agricultural University, Hisar - 125 004, India

**Keywords:** *Citrus sinensis*, Rutaceae, tetracosane, ethyl pentacosanoate, tetratriacontanoic acid, tangertin, β-sitosteryl-β-D-glucoside, 3,5,4'-trihydroxy-7,3'-dimethoxy flavanone 3-O-β-glucoside

## Abstract

Phytochemical examination of *Citrus sinensis* flavedo var. Pineapple resulted in the isolation of six compounds characterized as tetracosane, ethyl pentacosanoate, tetratriacontanoic acid, tangertin, β-sitosteryl-β-D-glucoside and 3,5,4'-trihydroxy-7,3'-dimethoxy flavanone 3-O-β-glucoside. Of these 3,5,4'-trihydroxy-7,3'-dimethoxy flavanone 3-O-β-glucoside is a hitherto unreported compound.

*Citrus sinensis* (L.) Osbeck var. Pineapple (syn, C. *aurantium* L. var. *sinensis)* belongs[1] to Rutaceae family and it is commonly known as sweet orange or *mosambi*. Its fruit is strengthening[2], cardiotonic, laxative, anthelmintic and removes fatigue. It possesses[3] antiinflammatory and antioxidant properties. A survey of literature showed that this variety had not been subjected to chemical analysis so far and the present investigation had therefore been undertaken.

Melting points were determined on Ganson Electrical Melting Point Apparatus. ^1^H NMR spectra were recorded on Bruker AC-400F MHz NMR Spectrometer in CDCI_3_ using TMS as internal standard. IR spectra were obtained on Perkin Elmer Infrared Spectrophotometer. Mass spectra were recorded on VG-70S 11-250J GC-MS-DS Mass Spectrometer.

Fruits (15 kg) of this plant were collected from Department of Horticulture, CCSHAU, Hisar. Flavedos (outer part of peel) were chopped into small pieces and then extracted with hot methanol. The solvent was removed to obtain extractives. The extractives were concentrated on a water bath under reduced pressure to obtain a viscous mass. The viscous mass thus obtained was mixed with silica gel (60-120 mesh), dried on water bath and subjected to silica gel (60-120 mesh) column chromatography and six compounds were isolated which were characterized as tetracosane (A), ethyl pentacosanoate (B), tetratriacontanoic acid (C), tangertin (D), β-sitosteryl-β-D-glucoside (E), 3,5,4'-trihydroxy-7,3'-dimethoxy flavanone 3-O-β-glucoside (Fig. 1). Of these, 3,5,4'-trihydroxy-7,3'-dimethoxy flavanone 3-O-β-glucoside is a hitherto unreported compound.

**Fig. 1:**
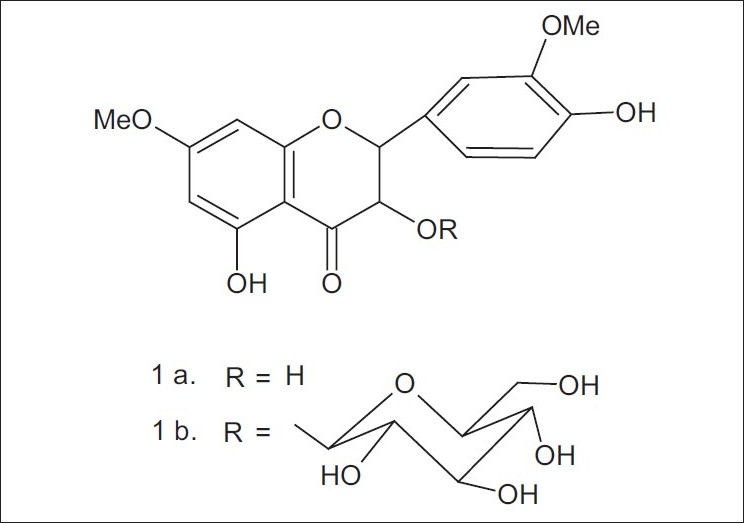
Structures of new compounds from Citrus sinensis 1a: 3,5,4'-trihydroxy-7,3'-dimethoxy flavanone, 1b: 3,5,4'-trihydroxy- 7,3'-dimethoxy flavanone 3-O-β-glucoside

Compound F (3,5,4'-trihydroxy-7,3'-dimethoxy flavanone 3-O-β-glucoside, (1b) obtained on elution with methanol-ethyl acetate (1:9) and it crystallized out from ethyl alcohol, 85 mg, m.p. 210°. It responded to Mg/HCl test. Alcoholic FeCl_3_ gave brown colour. Found 56.07, H 5.26; C_23_H_26_O_12_ requires C 55.87, H 5.26 %. UV λ_max_ (nm) 250, 287 (MeOH); 250, 287 (MeOH+NaOAc), 250, 311 (MeOH+AlCl_3_); 250, 311 (MeOH+AlCl_3_+HCl). IR ν_max_ (nujol, cm^−1^): 815, 975, 1070, 1095, 1131, 1202, 1278, 1358, 1443, 1518, 1607, 1647, 2918, 3471. MS (m/z): 494 (M^+^, 3), 464 (8), 463 (27), 446 (12), 418 (33), 386(36), 374 (41), 330 (100), 214 (38), 159 (5), 158 (16), 134 (3), 132 (10), 102 (6). Molish test: positive. Kiliani hydrolysis: glucose, confirmed by direct comparison using paper chromatography. Aglycone: UV λ_max_ (nm) 250, 288 (MeOH); 250, 288 (MeOH+NaOAc); 250, 310 (MeOH+AlCl_3_); 250, 310 (MeOH+AlCl_3_+HCl). Acetate of glycoside: Ac_2_O/Py, m.p. 144°. ^1^H NMR of glycoside acetate (δ, CDCl_3_): 2.04, 2.06, 2.07, 2.09 (3H each, 4s, 4*OAc); 2.32, 2.38 (3H each, 2s, 2*OAc); 2.70-5.50 (9 H, m, 2 C- ring protons, 7 glucose protons); 3.87 (6 H, s, 2*OMe); 6.31 (1H,*J* 2.5 Hz, H-6); 6.46 (1H, *J* 2.5 Hz, H-8); 7.01 (1H, *J* 7.5 Hz, H-5'); 7.15 (1H, d, *J* 2.5 Hz, H-2'); 7.26 (1H, dd, *J* 7.5 Hz, 2.5 Hz., H-6').

The brown colour with alcoholic ferric chloride indicated it to be a phenolic compound. The positive Mg/HCl colour reaction suggested it to be a flavonoid. IR depicted the presence of CO (1647 cm^−1^) and OH (3471 cm^−1^) groups. Elemental analysis indicated its molecular formula: C_23_H_26_O_12_ and molecular mass: 494. Its acetate was prepared by using acetic anhydride and pyridine. The ^1^H NMR of the acetate in CDCl_3_ showed a double doublet (*J* 7.5, 2.5 Hz) at δ 7.26, integrating to one proton, which could be H-6' of a flavanone. A doublet (*J* 2.5 Hz) was observed at δ 7.15, integrating to one proton, which could be H-2'. Another doublet (*J* 7.5 Hz.) was observed at δ 7.01, integrating to one proton, indicating it to be H-5'. There was a doublet (*J* 2.5 Hz.) at δ 6.46, integrating to one proton, which could be H-8. A doublet (*J* 2.5 Hz) at δ 6.31, for one proton, could be due to H-6. There was a singlet at δ 3.87, integrating to six protons, indicating two methoxy groups in the compound. There was a multiplet for nine protons in the range δ 2.70 - 5.50 and these could be seven protons of glucose and two protons of the C - ring of a flavanone. A singlet at δ 2.38, integrating to three protons, indicated a phenolic acetoxyl. Another singlet for second phenolic acetoxyl group was observed at δ 2.32. Four alcoholic acetoxyls at δ 2.09 (3H), 2.07(3H), 2.06 (3H), 2.04 (3H) indicated the presence of glucose in the molecule. This data suggested compound F to be 3,5,7,3',4'-pentaoxygenated flavanone-O-glucoside.

The original compound showed bathochromic shift with AlCl_3_/HCl hinting the presence of 5-OH in the compound F. The addition of sodium acetate did not show bathochromic shift in the UV-Vis of the glycoside and aglycone hinting the presence of 7-OMe in the both. The aglycone of the flavanone could be one of the following three, (i) 3,5,3'-trihydroxy-7,4'-dimethoxy (ii) 3,5,4'-trihydroxy-7,3'-dimethoxy (iii) 5,3',4'-trihydroxy-3,7-dimethoxy. The aglycone, obtained after Kiliani hydrolysis, had properties identical to (ii). The aglycone could therefore be settled as 3,5,4'-trihydroxy-7,3'-dimethoxy flavanone (1-a)[4].

The compound F could be one of the following two (i) 3,5,4'-trihydroxy-7,3'-dimethoxy flavanone 4'-O-glucoside (ii) 3,5,4'-trihydroxy-7,3'-dimethoxy flavanone-3-O-glucoside. The positions of H-5' in the acetates of compound F and that in 3,5,4'-trihydroxy-7,3'-dimethoxy flavanone are comparable; δ 7.01 in the former and δ 7.07 in the latter[4]; hinting that both have 4'-OH. The compound F could therefore be settled as 3,5,4'-trihydroxy-7,3'-dimethoxy flavanone 3-O-β-D- glucoside, (1 b).
